# Editorial: Advancing in the endoscopic ultrasound diagnosis of pancreatobiliary diseases

**DOI:** 10.3389/fmed.2023.1217977

**Published:** 2023-06-09

**Authors:** Pietro Fusaroli, Mamoru Takenaka, Yasunobu Yamashita

**Affiliations:** ^1^Department of Medical and Surgical Sciences, Gastrointestinal Unit, Hospital of Imola, University of Bologna, Bologna, Italy; ^2^Kindai University Hospital, Osaka-sayama, Japan; ^3^Second Department of Internal Medicine, Wakayama Medical University, Wakayama, Japan

**Keywords:** endosonography, pancreas, contrast—enhanced ultrasonography, elastography, screening

Endoscopic ultrasound (EUS) has entered its golden age. The benefits of providing refined diagnosis and advanced therapeutic procedures by EUS are numerous and widely appreciated by clinicians worldwide (1–3).

Just 20 years ago, EUS diagnosis was based mainly on radial mechanical echoendoscopes with no electronic image enhancement functions (4). On the other hand, electronic imaging including basic functions such as Doppler was available only for linear echoendoscopes, which were used mainly for EUS-guided tissue acquisition (5).

Since then, EUS has gone through years of exciting and unrelenting scientific and technologic advancements. The biggest improvements were introduced by image enhancement techniques with ultrasound contrast agents and elastography allowing better detection and characterization of the lesions of interest.

In this collection of *Frontiers in Medicine* about advancements in the EUS diagnosis of pancreatobiliary diseases, image enhanced EUS for the diagnosis of gallbladder lesions is presented. Differential diagnosis between benign and malignant gallbladder tumors can be challenging in order to select the candidates for surgery. Contrast-enhanced harmonic EUS (CH-EUS) has been previously reported to be useful for the diagnosis of gallbladder tumors (6). New and important findings are presented about the usefulness of EUS in the diagnosis of gallbladder polypoid lesions and gallbladder wall thickening. The characteristic findings of malignant gallbladder polypoid lesions on CH-EUS include irregular intratumoral vessels and perfusion defects such as hypovascular enhancement and inhomogeneous contrast distribution pattern. Staging is also aided by CH-EUS allowing the evaluation of the depth of invasion of the gallbladder wall.

The instance of rare pancreatic lesions that sometimes mimic other malignancies is also presented. Early detection and characterization of small and rare pancreatic malignancies is now possible by CH-EUS and EUS-elastography. A European multicenter study including undetermined small solid pancreatic lesions ≤15 mm in 394 asymptomatic patients demonstrated differential diagnosis between pancreatic ductal adenocarcinoma and tumors of different etiology by CH-EUS in 86% of the cases, with high diagnostic sensitivity (89%) and accuracy (90%) (7). Data in favor of EUS-elastography were reported by the same study group, which investigated solid pancreatic lesions ≤15 mm in comparison to the final diagnosis by EUS-guided tissue acquisition and/or surgery. High stiffness of the lesions had positive predictive value of 56% and a negative predictive value of 89% for the diagnosis of malignancy. For the diagnosis of pancreatic ductal adenocarcinoma, the sensitivity and specificity were 96 and 64%, respectively (8).

The usefulness of liquid-based cytology to increase the diagnostic yield of EUS-guided tissue acquisition is also discussed. Several techniques have been used in this regard, including fine needle biopsy with histology needles, rapid onsite cytopathology evaluation, and guidance by CH-EUS. A recent meta-analysis showed a pooled diagnostic sensitivity of 85% with CH-EUS-fine needle aspiration and 75% with standard EUS- fine needle aspiration (9). However, a recent trial from Taiwan that included patients investigated by EUS-fine needle biopsy and not EUS-fine needle aspiration failed to detect any benefit by CH-EUS guidance (10). It might be speculated that the use of histologic EUS needles can overcome the benefits of CH-EUS guidance to target the needle in specific area of pancreatic tumors.

EUS is also a great tool for pancreatic cancer screening in conjunction with magnetic resonance imaging (11). Interesting data are presented from a Japanese screening program using both techniques in high-risk individuals with a family history of pancreatic cancer. Interestingly, early abnormalities such as pancreatic cysts and mild EUS signs of chronic pancreatitis were useful for identifying curable pancreatic cancer.

Finally yet importantly, bibliometric analysis of EUS publications is presented. Compared to previous EUS literature scans (12), an update over the past 40 years shows that EUS has become a safe and effective tool for both diagnostic and therapeutic applications. Interestingly, the annual growth rate of publications from 1980 to 2020 was around 16% and the number of EUS-related articles had experienced a sudden increase in the last decade. Carcinoma, diagnosis, fine-needle-aspiration, cytology, and pancreatitis were the important keywords in co-occurrence analysis of keywords.

## Author contributions

PF wrote the editorial. MT and YY reviewed the manuscript for important intellectual content. All authors contributed to the article and approved the submitted version.
